# New Perspectives on the Role of Nuclear Proteases in Cell Death Pathways

**DOI:** 10.3390/biology12060797

**Published:** 2023-05-31

**Authors:** Anastasia S. Frolova, Olga E. Chepikova, Anna S. Deviataikina, Alena D. Solonkina, Andrey A. Zamyatnin

**Affiliations:** 1 Institute of Molecular Medicine, Sechenov First Moscow State Medical University, 119991 Moscow, Russia; frolanasta@gmail.com (A.S.F.); chepikovaolga@gmail.com (O.E.C.); 2 Scientific Center for Genetics and Life Sciences, Division of Biotechnology, Sirius University of Science and Technology, 354340 Sochi, Russia; 3 Institute of Biodesign and Complex Systems Modeling, Sechenov First Moscow State Medical University, 119435 Moscow, Russia; deviataikina.anna@mail.ru (A.S.D.); alene.s@mail.ru (A.D.S.); 4 Faculty of Bioengineering and Bioinformatics, Lomonosov Moscow State University, 119992 Moscow, Russia; 5 Belozersky Institute of Physico-Chemical Biology, Lomonosov Moscow State University, 119992 Moscow, Russia

**Keywords:** protease, nucleus, cell death, apoptosis, parthanatos, NETosis

## Abstract

**Simple Summary:**

For multicellular organisms, cell death is a vital process, which ultimately leads to the destruction of unneeded cells. Proteases are key enzymes that play a vital role in breaking down proteins and maintaining an optimal environment in the cell. During key stages of some types of cell death, the nucleus plays a critical role, and the activity of nuclear proteins determines the fate of the cell. Regulation of these proteins is crucial in determining whether the cell proceeds towards cell death or not. This review aims to provide a comprehensive picture of nuclear proteases that regulate and potentially participate in various types of cell death via the destruction of nuclear proteins.

**Abstract:**

Multiple factors can trigger cell death via various pathways, and nuclear proteases have emerged as essential regulators of these processes. While certain nuclear proteases have been extensively studied and their mechanisms of action are well understood, others remain poorly characterized. Regulation of nuclear protease activity is a promising therapeutic strategy that could selectively induce favorable cell death pathways in specific tissues or organs. Thus, by understanding the roles of newly discovered or predicted nuclear proteases in cell death processes, we can identify new pharmacological targets for improving therapeutic outcomes. In this article, we delved into the role of nuclear proteases in several types of cell death and explore potential avenues for future research and therapeutic development.

## 1. Introduction

Multicellular organisms are comprised of individual cells acting in tight and regulated cooperation. The development of an organism involves numerous rounds of cell division, but the number of cell divisions is limited, and as a result, the cell that gave rise to a large population dies. Cell death is the final stage in the life of a cell [1]. In some cases, cell death is delayed or, conversely, occurs prematurely. Tumor cells can avoid cell death, thereby replacing healthy tissues and migrating throughout the body [2]. In the case of neurodegenerative diseases, an increase in the occurrence of cell death is observed [3]. Therefore, a complete understanding of the mechanisms of cell death will provide an opportunity to find new options for the treatment of many diseases.

Every compartment in the cell has its own distinct function, with the nucleus serving as a crucial hub for genetic information storage and processing. The nucleus is responsible for controlling numerous cellular functions, including gene expression, DNA replication, and repair. One of the most significant changes that occurs during cell death is the alteration of chromatin structure, DNA degradation, and disassembly of nuclear structural proteins [4,5].

The destruction of nuclear and other proteins is accomplished by proteases, which hydrolyze peptide bonds between amino acids. Proteases are traditionally divided into specific groups according to the type of reaction they catalyze, amino acids in the active center, structure, and other features [6]. The key role of proteases is the ability to cleave proteins and thereby control individual protein levels. This allows proteases to participate in the regulation of many cellular processes, such as cell proliferation and differentiation [7], DNA replication, transcription and repair [8], angiogenesis and extracellular matrix remodeling [9,10], immunity [11,12], cell death [13], etc. Proteases are also involved in pathological conditions. In cancer, proteases regulate proteolytic extracellular matrix (EMC) remodeling, altering cell–cell and cell–matrix interactions that facilitate invasion and metastasis [14,15,16,17,18].

Proteases have been identified in the nucleus [19,20,21]. These proteases play a significant role in the regulation of gene expression [22,23], as well as in the immune response, carcinogenesis [24], and cell death [25]. Recent research has revealed that proteases in the nucleus perform a variety of functions, which include processing and activation of transcription factors, chromatin remodeling, histone modification, and DNA repair. Proteases also play a role in maintaining the structural integrity of the nucleus. The activity of nuclear proteases is tightly regulated to ensure the proper functioning of the cell. Dysregulation of these enzymes can lead to a wide range of diseases, including cancer or neurodegenerative disorders.

While the core machinery of cell death mechanisms is known, novel participants in various pathways are constantly being identified. In this review, we focus on cell death mechanisms that involve the nucleus and the involvement of nuclear proteases in these processes.

## 2. Nuclear Proteases

Proteases participate in many cellular processes, in which it is necessary to cleave proteins in the extracellular matrix, on the cellular membrane or in some cellular compartments. In many cases, proteases have either a specific localization, such as on the membrane [26], or their localization in cells change due to different factors [27]. In this review, we assign certain localizations to proteases based on the compartments where they function during certain stages of a cell’s lifetime.

Proteases that are secreted into the extracellular environment or anchored in the cell membrane can affect morpho- and angiogenesis by remodeling the extracellular matrix [9,10]. Acidic vesicles, known as lysosomes, contain pH-dependent proteases—cathepsins, napsins, and asparagine endopeptidase [28]. These lysosomal proteases are responsible for protein degradation during phagocytosis, endocytosis, and autophagy, but are also involved in growth factor signaling, and antigen presentation. Mitoproteases, which are found in mitochondria, degrade misfolded or damaged proteins, regulate mitochondrial gene expression and mitophagy, and activate or inhibit a number of other pathways [28].

Proteases can also be found in the nucleus of normal [29], as well as cancer cells [30,31,32,33,34,35]. In this review, we focus on nuclear proteases that reside in the nucleus at a given time and are responsible for the degradation of nuclear proteins or regulating nuclear processes within cells. We are not asserting that the nuclear proteases outlined in this review are exclusively nuclear proteins. It was found that nuclear proteases degrade different substrates involved in the cell cycle [36], DNA repair [37], cell senescence [38], and carcinogenesis [39,40].

Research indicates that nuclear proteases may exhibit high specificity towards nuclear proteins. For instance, nuclear cathepsins have been observed to cleave specific nuclear substrates, while lysosomal cathepsins can cleave all available proteins [41]. This specificity presents an exciting opportunity to use nuclear proteases for cleaving specific proteins, potentially modulating cell death by degrading key proteins. Furthermore, nuclear proteases might be utilized for the degradation of unwanted accumulated proteins, such as polyglutamine proteins that are associated with neuropathology [42]. Targeted degradation of these proteins using nuclear proteases could be a promising approach for treatment. Studying the nuclear substrates of nuclear proteases could also provide insight into utilizing them in targeting systems such as PROTACs (proteolysis targeting chimeras) for specific degradation of nuclear proteins [43]. The potential applications of nuclear proteases are vast, but careful study of these proteins is required to fully understand their functions and potential. Understanding the mechanism of action of nuclear proteases, their specificity, and the consequences of their activity will be critical in developing effective therapies and treatments that can harness their potential. Future research should continue to explore the role of nuclear proteases in cellular processes and their potential applications in therapeutic interventions.

Many proteases exhibit both nuclear and cytoplasmic localization, and it remains unclear why certain proteases translocate into the nucleus. Detailed analysis of protein sequences has revealed that some proteases possess a nuclear translocation signal (NLS). For example, matrix metalloproteinase-2 (MMP-2) has an NLS on its C-terminus, and amino acid substitutions in this region result in loss of nuclear localization [44]. Bioinformatic analysis of MMP proteins has shown that all members of this group contain one or more NLSs [21]. However, not all of these proteases have been found in the nucleus, indicating that the presence of an NLS alone may not be sufficient for nuclear translocation. The localization of the NLS within the protein structure plays an important role in determining its localization. In some cases, the NLS may be masked by a prodomain or linker region, preventing the protein from translocating into the nucleus [25]. Given these findings, the mechanisms that dictate whether proteases in their active or inactive forms exhibit nuclear localization are still not fully understood. Further research is necessary to elucidate the factors that influence nuclear translocation of proteases, which could have significant implications for understanding their regulation.

## 3. Nuclear Compartment in Cell Death

The nucleus is a defining feature of eukaryotic cells and is the largest organelle in most cells. It separates the genome and transcriptional machinery from the cytoplasm [45]. The nucleus serves as the cell’s control center by coordinating processes such as cell growth, metabolism, and cell division. In addition, the nucleus plays a role in some forms of cell death.

There are different classifications and nomenclatures of cell death, based on multiple mechanisms and phenotypes. Historically, three morphologically distinct categories (type I-III cell death), namely apoptosis, autophagy, and necrosis, have been used for classification [46,47]. This morphological classification is still extensively employed. In 2018, the Nomenclature Committee on Cell Death provided molecular marker-based definitions of cell death types. Intrinsic and extrinsic apoptosis are types of cell death that occur in response to internal or external signals. Mitochondrial permeability transition (MPT)-driven necrosis is caused by mitochondrial destruction, while necroptosis and parthanatos are mediated by specific proteins. Iron overload and lipid peroxidation are the triggers for ferroptosis, whereas pyroptosis, entotic cell death, NETosis, and immunogenic cell death are types of cell death that occur as a consequence of an inflammatory response. Two types of cell death involve specific compartments harboring various proteases, namely lysosome-dependent cell death and autophagy-dependent cell death. Cellular senescence is a form of cell death that occurs due to a state of cell division arrest, and mitotic catastrophe happens when cells perform an abortive act of cell division. Cell death can happen in two ways: due to overwhelming damage, which is called accidental cell death, or as a result of specific signaling events, also known as regulated cell death (RCD), which is the physiological form of programmed cell death [48,49]. Among all the mentioned types of cell death, only three occur with the participation of the cell nucleus: apoptosis, parthanatos, and NETosis.

Apoptosis is a multi-pathway mode of cell death that leads to the destruction of cells and the nucleus plays a crucial role in this process. The intrinsic pathway of apoptosis is initiated by internal signals, including DNA damage, which triggers the release of cytochrome *c* from mitochondria. The regulation of this pathway is carried out by pro- and anti-apoptotic proteins of the BCL-2 family, as well as initiator and effector caspases [50,51]. The activation of initiator caspases by cytochrome *c* in turn activates the main effector caspases. In contrast, extrinsic signals activate a distinct apoptosis pathway, which ultimately leads to the activation of effector caspases. Once translocated into the nucleus, effector caspases cleave several nuclear proteins, including poly(ADP-ribose) polymerase-1 (PARP-1), lamin, β-tubulin, and others [52]. Cleavage of the inhibitor of caspase-activated DNase (ICAD) by caspase-3 is a crucial event in the apoptotic pathway, allowing caspase-activated DNase (CAD) to induce oligonucleosomal DNA fragmentation [53,54,55]. Other mitochondrial proteins, such as endonuclease G (EndoG) and apoptosis-inducing factor (AIF), also enter the nucleus and initiate chromatin condensation and DNA fragmentation, which can later lead to membrane blebbing. In the final stages of apoptosis, the cell partitions into small apoptotic bodies that are eliminated by macrophages or surrounding cells. In that case, the contents of the cell is not released into the environment and does not trigger an inflammatory reaction.

Parthanatos is a distinct type of cell death that is mainly triggered by DNA damage. In response to this, PARP-1 protein begins to produce an excessive amount of poly(ADP-ribose) (PAR), which is then translocated into the mitochondria. The PAR molecules interact with the mitochondria, inducing the release of AIF [56]. Once AIF enters the nucleus, it triggers extensive DNA fragmentation and chromatin condensation, ultimately leading to cell death. The translocation of AIF from mitochondria to the nucleus, and subsequent nucleus destruction, which is characteristic of this type of cell death, highlights the critical role of the nucleus in parthanatos [57].

NETotic cell death is thought to involve a complex signaling pathway [58,59,60]. Activation of NADPH oxidase by chemical reagents or bacterial action leads to the formation of reactive oxygen species (ROS) [58]. The presence of ROS triggers the release of bactericidal proteins, such as antimicrobial peptides, cytokines, and digestive enzymes, including neutrophil elastase (NE), cathepsin G, azurocidin, and myeloperoxidase (MPO), from the azurophilic granules of neutrophils into the cytosol [61]. NE partially translocates into the nucleus and cleaves nuclear proteins [62]. Peptidyl arginine deiminase 4 (PAD4) also enters the nucleus, where it induces histone citrullination [63,64], leading to DNA decondensation. In the next stage of NETosis, decondensed chromatin, decorated with histones and antimicrobial proteins, is released into the cytoplasm as a result of rupturing of the nuclear envelope. This forms a net-like structure, termed the neutrophil extracellular trap (NET), which is then expelled from the cell [58]. The nucleus’s involvement in NETotic cell death underscores the significance of this compartment in cell death mechanisms.

**Figure 1:**
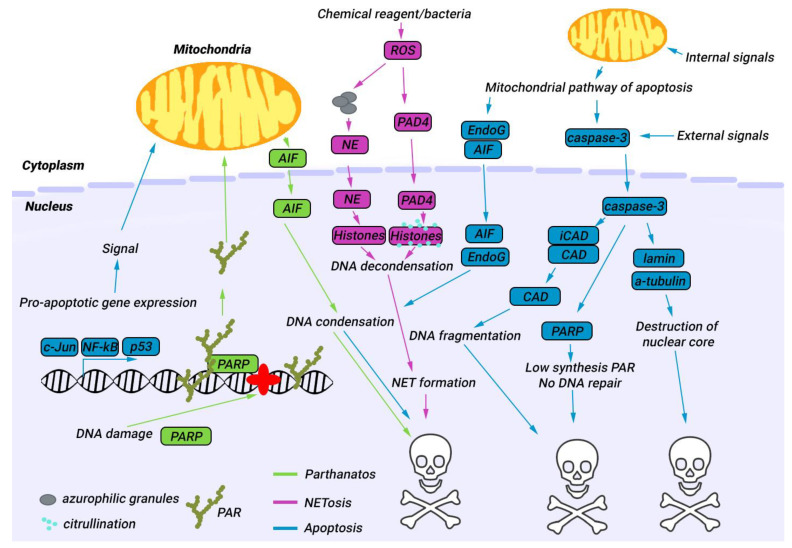
Role of nucleus in three types of cell death: apoptosis, NETosis and parthanatos. Although these cell death pathways have different activators, the nuclear events that occur during these processes are similar. This includes the degradation of structural and functional proteins by proteases, as well as DNA decondensation or degradation by endonucleases or modification enzymes. Apoptosis proteins are indicated in blue boxes; NETosis—pink, parthanatos—green.

The cell death mechanisms in the nucleus share a similar pattern across the three types of cell death: apoptosis, NETosis, and parthanatos (Figure 1). During apoptosis and parthanatos, DNA condensation occurs through the common protein AIF, while NETosis involves DNA decondensation, which is critical for NET formation. DNA fragmentation is exclusive to apoptosis. These three cell death types also involve disruption of the nuclear envelope to a varying degree. For example, in apoptosis, nuclear proteases cleave lamins, which leads to destruction of the nuclear envelope and the nucleus as a whole [65,66]. During NETosis, pores form in the nuclear envelope, possibly due to the insertion of gasdermin D protein into the membrane [67]. Disassembly of nuclear lamin without proteolysis is also observed [68]. The process of nuclear destruction during parthanatos has not been extensively studied, and the proteins that are responsible for this process remain unknown.

All these processes involve important nuclear regulatory proteins in the cell nucleus. Apoptosis demonstrates how the degradation of such proteins by nuclear proteinases can regulate cell death (Table 1). The pathways of NETosis and parthanatos are not yet fully understood, and there are many gaps in our knowledge of the nucleus’s role in these processes that may involve the action of nuclear proteases.

## 4. Nuclear Proteases in Apoptosis

Apoptotic cell death is associated with proteolytic processes [93]. The main proteases involved in apoptosis are caspases [94]. Upon activation, caspases cleave and activate pro-apoptotic or structural proteins, the destruction of which is necessary for cell death [95]. Caspases that are translocated into the nucleus degrade nuclear lamins [65], importin-α [71], large subunit of the DNA replication complex C [72], ICAD [54,74], Rad51 [73], PARP [70], and other nuclear proteins. Therefore, at a physiological level, cells experience rounding, chromatin condensation, DNA fragmentation, blebbing of membrane, and as a result—formation of apoptotic bodies [96].

Although caspases play a significant role in apoptosis, other cellular proteases also contribute to it. For instance, during H_2_O_2_-induced apoptosis, cathepsins L and B, but not calpains, participate in caspase activation and DNA fragmentation [97]. This is related to the activity of these proteases in the cytoplasm, but the diversity of proteases in the nucleus suggests the presence of currently unknown participants in apoptosis. Experiments with lysates from Fas-stimulated Jurkat cell and isolated nuclei have shown that these lysates contain serine proteases that induce DNA fragmentation in an apoptosis-dependent manner [98,99]. In another experiment, the addition of Ca^2+^ to isolated nuclei initiated the fragmentation of DNA into small fragments (50 kbp), which were previously observed in apoptotic nuclei, and this depended on the activity of a serine protease and calpain [100].

Calpains are a group of non-caspase Ca^2+^-dependent proteases that are activated when the intracellular Ca^2+^ level increases and they play a specific role in neuronal apoptosis [101]. Calpains translocated into the nucleus are involved in DNA fragmentation in Ca^2+^- treated nuclei [100]. In vitro and in vivo analysis of maitotoxin-treated cells revealed that PARP-1 protein is cleaved by a calpain, which leads to the formation of a 40 kDa immunoreactive fragment [82].

Another participant of apoptotic nuclear events is the serine protease granzyme. Granzymes are located in cytotoxic granules of immune cells and are secreted into the extracellular matrix for the elimination of target cells [102]. These proteases contribute to apoptotic pathways through caspase activation and directly induce protein degradation in the nucleus [103]. It was found that under caspase-inhibited conditions, cells undergo apoptosis because of granzyme-dependent cleavage of nuclear lamins, PARP, ICAD [78,79,80], and activation of DNA fragmentation [81].

Transcriptional factors play an important role in the regulation of gene expression and apoptosis [104]. An alternative way to control the activation or the deactivation of regulator pathways is the degradation of transcription factors with nuclear proteases. Transcription factor Yin Yang 1 (YY1) is degraded by nuclear cathepsin-B-like protease in NT2 cells after treatment with retinoic acid [105]. In pancreatic cancer cells, YY1 activates transcription of pro-apoptotic Bax protein and thereby facilitates apoptosis [106]. Another nuclear cathepsin-B-like protease, referred to as SPase, cleaves transcriptional factor Sp1 and RB proteins in CV-1 cells. Different studies have revealed that Sp1 protein is involved in the regulation of apoptosis [107], and during DNA-induced apoptosis Sp1 is cleaved by caspase-3 [69].

Nuclear cathepsins also have some nuclear substrates that participate in apoptosis. Recent studies have identified several of these, including the transcriptional factor p53 and prohibitin [76]. The inhibition of cathepsin L in U87 glioblastoma cells has been shown to have a significant impact on the accumulation of p53 and prohibitin in the cell nucleus. This suggests that cathepsin L plays a unique role in the regulation of transcription of caspase-3 and caspase-7, which are key players in apoptotic cell death. Apart from the nuclear cathepsins, cathepsin B is another protease that has been found to have apoptogenic activity [77]. The treatment of the nucleus from digitonin-permeabilized cells with purified cathepsin B leads to DNA condensation and fragmentation after just 15 min. These features are characteristic of apoptosis, suggesting that cathepsin B plays a significant role in this process.

## 5. Nuclear Proteases in Parthanatos

Parthanatos is activated in the nucleus during DNA damage [57]. Overactivated PARP-1 produces PARs in response to DNA damage. PARP-1 is a main member of the PARP family and accounts for 90% of the activity of these proteins. It is a nuclear enzyme that activates in response to DNA strand breaks and forms linear or branched PARs. These PARs are then linked with PARP, histones, DNA helicases, topoisomerases, single-strand break repair factors, base-excision repair factors and several transcription factors [108]. During the overproduction of PAR, and despite the activation of the DNA repair system, effective DNA repair does not occur, and cell death is potentiated due to repair-induced DNA decondensation. The excess PARs partially translocate from the nucleus to mitochondria, leading to the release and transfer of AIF protein into the nucleus [57]. AIF is a FAD-dependent oxidoreductase that accumulates and induces peripheral DNA condensation and large DNA fragmentation [56].

**Figure 2:**
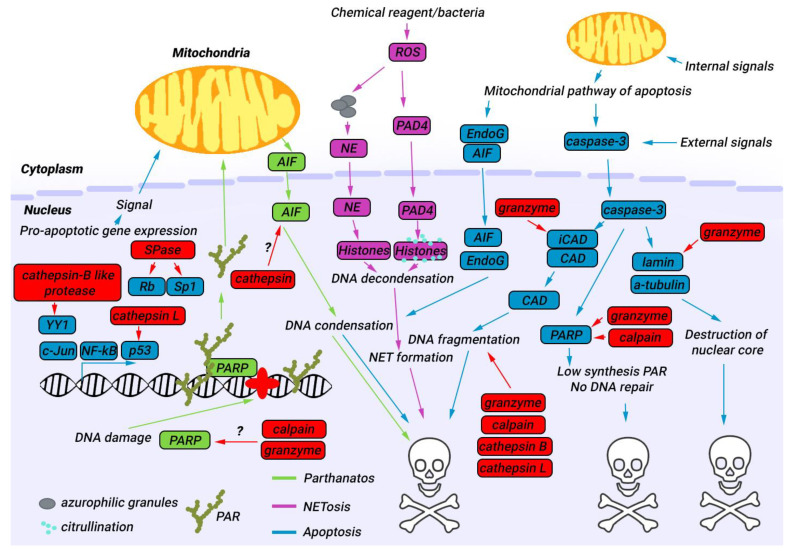
The involvement of nuclear proteases in cell death. Understudied nuclear proteases are capable of degrading a wide range of critical nuclear proteins, including PARP, lamin, and various transcriptional factors. However, the specific mechanisms by which some of these nuclear proteases contribute to cell death remain unknown. Proteins associated with apoptosis are indicated in blue boxes; NETosis—pink, parthanatos—green. Nuclear proteases are indicated in red boxes.

Among the main participants in apoptosis, no nuclear proteases were found, although some of the proteins undergo degradation during other types of cell death (Figure 2). For instance, polymerase PARP-1 degrades during caspase-dependent apoptosis [70]. In caspase-independent apoptosis, calpain [82] and granzyme [80] can also cleave PARP-1. Hyperactivation of PARP-1 can be blocked with a specific PARP inhibitor [109]. On the other hand, the activity of the protein can be abrogated via its degradation by nuclear proteases. The main question is how these nuclear proteases can be activated.

Another potential substrate for nuclear proteases is nuclear AIF. It was shown that cysteine proteases are involved in the degradation of intracellular AIF [92]. Based on the fact that some cysteine proteases, such as cathepsins [76], are present in cell nuclei, it can be assumed that under certain conditions, these nuclear proteases can potentially degrade AIF.

## 6. Nuclear Proteases in NETosis

NETosis is a specific form of cell death primarily observed in neutrophils but also reported in other leukocytes [110,111,112,113]. Activation of signaling pathways induces the production of ROS, chromatin decondensation, and the release of NETs [58,114].

Neutrophil elastase is a serine protease stored in azurophilic granules that is involved in antimicrobial activity in the phagosome [115]. During NETosis, after translocation into the nucleus from cytoplasm, NE participates in the cleavage of histones that maintain chromosome structure [116]. It has been shown that NE is essential for NET formation and DNA decondensation via its involvement in the destruction of core histones H2A, H2B, H3, H3, and linker histone H1 [62]. In the case of the H2A histone, neutrophil elastase cleaves the protein at position V_114_ [91]. As a result, histone degradation promotes chromosome decondensation and NETs formation.

In NETosis, calpain synergizes with PAD4, and can participate in this type of cell death only in this synergistic manner. The nucleus, previously treated with PAD4, expands, undergoes chromatin decondensation, and forms NETs in the presence of calpain [90]. Protein analysis of treated nuclei show that lamin A/C, as well as nuclear protein HMGB1, N-terminus of H3 histone, and HP1a undergo calpain-mediated proteolysis.

The function of other neutrophil serine proteases (NSP) remains unclear. Kasperkiewicz et al. revealed that the catalytic activity of cathepsin G, proteinase 3, and NSP4 is not required for NETs formation [117]. Isolated neutrophils were treated with specific inhibitors of NSP, and NETosis was induced with different stimuli (PMA, LPS or bacteria). Inhibition of cathepsin G, proteinase 3, and NSP4 blocked the release of DNA. Additionally, these serine proteases are present in NET structures in their inactive form.

Thus, during NETosis, the primary role of nuclear proteases is to induce or facilitate the process of chromosome decondensation, which is a critical step in the subsequent stages of NETs formation. Understanding the underlying mechanisms of NETosis and its regulation are crucial for developing effective treatments for a variety of diseases, including autoimmune disorders and infectious diseases.

## 7. Approaches for Modulating the Activity of Proteases in the Nucleus

Misregulation of cell death mechanisms can result in various consequences, such as uncontrolled cell division and proliferation, as observed in tumor cells [2], or excessive cell death, as seen in the case of neural cells in neurodegenerative diseases [3]. Thus, inducing or inhibiting cell death can be a promising approach to treating highly relevant diseases. Targeting specific proteins involved in the cell death pathways is one of the ways to control cell death. Importantly, nuclear proteases are potential targets for regulating cell death. However, it is crucial to note that while targeting nuclear proteases can be effective, it may also have unintended consequences as these proteases may have other functions in cells. Further research is needed to understand the potential risks and benefits of targeting nuclear proteases in regulating cell death.

Nuclear proteases play a specific role in different stages of cell death, and targeting a certain protease can either activate or inhibit cell death specifically. However, uncontrolled action of proteases can lead to cell death, which is why cells contain specific endogenous inhibitors, for example, MMP and tissue inhibitors of metalloproteinase (TIMPs) [118], cathepsin and cystatin [119], caspase and its inhibitors [120], calpain and calpastatin [121], and others. It is worth noting that both proteases and their inhibitors are present in the cell nucleus [122,123,124]. However, endogenous inhibitors are distributed throughout the cell, and inhibition of a particular nuclear protease can also affect its activity in the cytoplasm. One potential solution is to create a specific nuclear inhibitor that works only in the nucleus. While this approach is still under investigation, it has the potential to provide more targeted and precise regulation of cell death. Further research is necessary to understand the potential risks and benefits of creating specific nuclear inhibitors and how they might impact cellular processes beyond cell death.

Numerous proteases are produced in an inactive form. Their maturation involves cleavage of a prodomain, which inhibits protease activity. This process can be accomplished by other proteases [125] or autoactivation [126]. However, the mechanisms by which proteases are activated in the nucleus or translocated into the nucleus in response to different stimuli is not yet fully understood. The lack of information on this topic limits our ability to create unique and specific activators for nuclear proteases. Despite this, common practice is to use activators for proteases in the whole cell to induce sufficient cell death [127,128]. Targeting nuclear proteases could be a more effective approach in regulating cell death compared to others. However, further research is necessary to understand the mechanisms of action of nuclear proteases and their potential as targets for regulating cell death.

## 8. Conclusions

Proteases are a vast class of proteins that play a crucial role in various cellular processes, including homeostasis, tissue development, angiogenesis, cell death, autophagy, immune response, DNA repair, replication, transcription, and many others. In the context of cell death, proteases are particularly important, as exemplified by caspases in apoptosis. In this review, we have described the role of nuclear proteases in apoptosis, NETosis, and parthanatos, highlighting the general mechanisms of these cell death pathways with detailed descriptions of nuclear protease involvement.

Further research is needed to fully understand the mechanisms of action of nuclear proteases in cell death and their potential as therapeutic targets. By understanding the specific roles of nuclear proteases in cell death pathways, it may be possible to develop specific inhibitors or inducers of these proteases, potentially leading to new treatments for diseases associated with aberrant cell death processes.

## Figures and Tables

**Table 1:** Nuclear substrates in apoptosis, parthanatos, and NETosis.

Cell Death	Protease	Substrate in Nucleus	Substrate Cell Function	What Happened after Cleavage	Ref.
apoptosis	caspase-3	Sp1	Transcription factor	Apoptosis	[69]
		PARP-1	DNA repair	Activation of apoptosis	[70]
		lamin	Nuclear envelope	Degradation of nucleus	[65]
		importin-α	Import of protein in cell nucleus	Downregulate DNA synthesis	[71]
		large subunit of the DNA replication complex C	Regulation of DNA replication	Decrease DNA binding	[72]
		Rad51	DNA repair	Activation of apoptosis	[73]
		ICAD	Inhibition of CAD	DNA fragmentation	[54,74]
	calpain	lamin A	Nuclear envelope	Degradation of nucleus	[66]
		lamin B	Nuclear envelope	Degradation of nucleus	[66]
		spectrin	Skeletal protein	Product of SBPD145, 150i, 120 Activation of apoptosis	[75]
	cathepsin L	p53	Transcription factor, regulation of caspase-7 expression	Silencing of CtsL induce the decrease in p53	[76]
		prohibitin	Transcription factor, regulation of caspase-7 expression	Silencing of CtsL induce the decrease in p53	[76]
	cathepsin B	-	-	DNA condensation and fragmentation	[77]
	granzyme	lamin	Nuclear envelope	-	[78]
		PARP	DNA repair	-	[79,80]
		ICAD	Inhibition of CAD	-	[79]
		-	-	DNA fragmentation	[81]
?—apoptosis	calpain	PARP	DNA repair	-	[82]
		CaMK4	Calcium signaling, regulates β-cell apoptosis	-	[83]
		β-catenin	Transcription factor, regular expression of Wnt pathways genes	-	[84]
		c-Fos	Transcription factor	-	[85,86]
		c-Jun	Transcription factor	-	[85,86]
		Sp3, Sp4	Transcription factor	-	[87]
		p53	Transcription factor	-	[88]
	SPase	Sp1	Transcription factor	-	[89]
		Rb	Regulates cell growth	-	[89]
NETosis	calpain	H3	Maintains structure of DNA	Degradation of nuclear envelope	[90]
		HP1a	Gene regulation	Chromatin decondensation	[90]
		lamin A/C	Nuclear core structure	Degradation of nuclear envelope	[90]
		H3	Maintains structure of DNA	Degradation of nuclear envelope	[90]
		?	?	Chromatin decondensation	[90]
	neutrophil elastase	H1, H2A, H2B, H3, H3	Maintains structure of DNA	Chromatin decondensation	[62,91]
?—parthanatos	calpain	PARP	-	-	[82]
	granzyme	PARP	-	-	[79]
	cysteine protease/cathepsin	AIF	-	-	[76,92]

?—We can speculate the involvement in cell death based on the substrate.

## Data Availability

Not applicable.

## References

[1] Gudipaty S.A., Conner C.M., Rosenblatt J., Montell D.J. (2018). Unconventional Ways to Live and Die: Cell Death and Survival in Development, Homeostasis, and Disease. Annu. Rev. Cell Dev. Biol..

[2] Fernald K., Kurokawa M. (2013). Evading apoptosis in cancer. Trends Cell Biol..

[3] Cui J., Zhao S., Li Y., Zhang D., Wang B., Xie J., Wang J. (2021). Regulated cell death: Discovery, features and implications for neurodegenerative diseases. Cell Commun. Signal..

[4] Prokhorova E.A., Zamaraev A.V., Kopeina G.S., Zhivotovsky B., Lavrik I.N. (2015). Role of the nucleus in apoptosis: Signaling and execution. Cell. Mol. Life Sci..

[5] Saunders C.A., Parent C.A. (2019). ScienceDirect Emerging roles for the nucleus during neutrophil signal relay and NETosis. Curr. Opin. Cell Biol..

[6] Gurumallesh P., Alagu K., Ramakrishnan B., Muthusamy S. (2019). A systematic reconsideration on proteases. Int. J. Biol. Macromol..

[7] King R.W., Deshaies R.J., Peters J.-M., Kirschner M.W. (1996). How Proteolysis Drives the Cell Cycle. Science.

[8] Burby P.E., Simmons Z.W., Schroeder J.W., Simmons L.A. (2018). Discovery of a dual protease mechanism that promotes DNA damage checkpoint recovery. PLoS Genet..

[9] Ghajar C.M., George S.C., Putnam A.J. (2008). Matrix Metalloproteinase Control of Capillary Morphogenesis. Crit. Rev. Eukaryot. Gene Expr..

[10] Lakka S.S., Gondi C.S., Rao J.S. (2005). Proteases and glioma angiogenesis. Brain Pathol..

[11] Qureshi N., Vogel S.N., Van Way C., Papasian C.J., Qureshi A.A., Morrison D.C. (2005). The Proteasome: A Central Regulator of Inflammation and Macrophage Function. Immunol. Res..

[12] Lamkanfi M., Kanneganti T.D. (2010). Caspase-7: A protease involved in apoptosis and inflammation. Int. J. Biochem. Cell Biol..

[13] Martin S.J. (2014). Caspases: Executioners of Apoptosis. Pathobiology of Human Disease.

[14] Tan G.-J., Peng Z.-K., Lu J.-P., Tang F.-Q. (2013). Cathepsins mediate tumor metastasis. World J. Biol. Chem..

[15] Tedelind S., Jordans S., Resemann H., Blum G., Bogyo M., Führer D., Brix K. (2011). Cathepsin B trafficking in thyroid carcinoma cells. Thyroid Res..

[16] Mohamed M.M., Sloane B.F. (2006). Cysteine cathepsins: Multifunctional enzymes in cancer. Nat. Rev. Cancer.

[17] Sloane B.F., Rozhin J., Johnson K., Taylor H., Crissman J.D., Honn K.V. (1986). Cathepsin B: Association with plasma membrane in metastatic tumors. Proc. Natl. Acad. Sci. USA.

[18] Egeblad M., Werb Z. (2002). New functions for the matrix metalloproteinases in cancer progression. Nat. Rev. Cancer.

[19] Mannello F., Medda V. (2012). Nuclear localization of Matrix metalloproteinases. Prog. Histochem. Cytochem..

[20] Soond S.M., Kozhevnikova M.V., Frolova A.S., Savvateeva L.V., Plotnikov E.Y., Townsend P.A., Han Y.P., Zamyatnin A.A. (2019). Lost or Forgotten: The nuclear cathepsin protein isoforms in cancer. Cancer Lett..

[21] Frolova A.S., Petushkova A.I., Makarov V.A., Soond S.M., Zamyatnin A.A. (2020). Unravelling the network of nuclear matrix metalloproteinases for targeted drug design. Biology.

[22] Sounni N.E., Roghi C., Chabottaux V., Janssen M., Munaut C., Maquoi E., Galvez B.G., Gilles C., Frankenne F., Murphy G. (2004). Up-regulation of Vascular Endothelial Growth Factor-A by Active Membrane-type 1 Matrix Metalloproteinase Through Activation of Src-Tyrosine Kinases. J. Biol. Chem..

[23] Burton L.J., Dougan J., Jones J., Smith B.N., Randle D., Henderson V., Odero-Marah V.A. (2017). Targeting the Nuclear Cathepsin L CCAAT Displacement Protein/Cut Homeobox Transcription Factor-Epithelial Mesenchymal Transition Pathway in Prostate and Breast Cancer Cells with the Z-FY-CHO Inhibitor. Mol. Cell. Biol..

[24] Xie Y., Lu W., Liu S., Yang Q., Shawn Goodwin J., Sathyanarayana S.A., Pratap S., Chen Z. (2016). MMP7 interacts with ARF in nucleus to potentiate tumor microenvironments for prostate cancer progression in vivo. Oncotarget.

[25] Si-Tayeb K., Monvoisin A., Mazzocco C., Lepreux S., Decossas M., Cubel G., Taras D., Blanc J.F., Robinson D.R., Rosenbaum J. (2006). Matrix metalloproteinase 3 is present in the cell nucleus and is involved in apoptosis. Am. J. Pathol..

[26] Knapinska A.M., Fields G.B. (2019). The expanding role of mt1-mmp in cancer progression. Pharmaceuticals.

[27] Zheng T.S., Schlosser S.F., Dao T., Hingorani R., Crispe I.N., Boyer J.L., Flavell R.A. (1998). Caspase-3 controls both cytoplasmic and nuclear events associated with Fas-mediated apoptosis in vivo. Proc. Natl. Acad. Sci. USA.

[28] Müller S., Dennemärker J., Reinheckel T. (2012). Specific functions of lysosomal proteases in endocytic and autophagic pathways. Biochim. Biophys. Acta—Proteins Proteom..

[29] Malara A., Ligi D., Di Buduo C., Mannello F., Balduini A. (2018). Sub-Cellular Localization of Metalloproteinases in Megakaryocytes. Cells.

[30] Tedelind S., Poliakova K., Valeta A., Hunegnaw R., Yemanaberhan E.L., Heldin N.E., Kurebayashi J., Weber E., Kopitar-Jerala N., Turk B. (2010). Nuclear cysteine cathepsin variants in thyroid carcinoma cells. Biol. Chem..

[31] Puchi M., García-Huidobro J., Cordova C., Aguilar R., Dufey E., Imschenetzky M., Bustos P., Morin V. (2010). A new nuclear protease with cathepsin L properties is present in HeLa and Caco-2 cells. J. Cell. Biochem..

[32] Tamhane T., Wolters B.K., Illukkumbura R., Maelandsmo G.M., Haugen M.H., Brix K. (2015). Construction of a plasmid coding for green fluores protein tagged cathepsin L and data on expression in colorectal carcinoma cells. Data Br..

[33] Bestvater F., Dallner C., Spiess E. (2005). The C-terminal subunit of artificially truncated human cathepsin B mediates its nuclear targeting and contributes to cell viability. BMC Cell Biol..

[34] Hiwasa T., Sakiyama S. (1996). Nuclear localization of procathepsin L/MEP in ras-transformed mouse fibroblasts. Cancer Lett..

[35] Sullivan S., Tosetto M., Kevans D., Coss A., Wang L., O’Donoghue D., Hyland J., Sheahan K., Mulcahy H., O’Sullivan J. (2009). Localization of nuclear cathepsin L and its association with disease progression and poor outcome in colorectal cancer. Int. J. Cancer.

[36] Goulet B., Truscott M., Nepveu A. (2006). A novel proteolytically processed CDP/Cux isoform of 90 kDa is generated by cathepsin L.. Biol. Chem..

[37] Hill J.W., Poddar R., Thompson J.F., Rosenberg G.A., Yang Y. (2012). Intranuclear matrix metalloproteinases promote DNA damage and apoptosis induced by oxygen-glucose deprivation in neurons. Neuroscience.

[38] Duarte L.F., Young A.R.J., Wang Z., Wu H.A., Panda T., Kou Y., Kapoor A., Hasson D., Mills N.R., Ma’ayan A. (2014). Histone H3.3 and its proteolytically processed form drive a cellular senescence programme. Nat. Commun..

[39] Ip Y.C., Cheung S.T., Fan S.T. (2007). Atypical localization of membrane type 1-matrix metalloproteinase in the nucleus is associated with aggressive features of hepatocellular carcinoma. Mol. Carcinog..

[40] Mäkinen L.K., Häyry V., Atula T., Haglund C., Keski-Säntti H., Leivo I., Mäkitie A., Passador-Santos F., Böckelman C., Salo T. (2012). Prognostic significance of matrix metalloproteinase-2, -8, -9, and -13 in oral tongue cancer. J. Oral Pathol. Med..

[41] Stratigopoulos G., LeDuc C.A., Cremona M.L., Chung W.K., Leibel R.L. (2011). Cut-like homeobox 1 (CUX1) regulates expression of the fat mass and obesity-associated and retinitis pigmentosa GTPase regulator-interacting protein-1-like (RPGRIP1L) genes and coordinates leptin receptor signaling. J. Biol. Chem..

[42] Havel L.S., Li S., Li X.-J. (2009). Nuclear accumulation of polyglutamine disease proteins and neuropathology. Mol. Brain.

[43] Békés M., Langley D.R., Crews C.M. (2022). PROTAC targeted protein degraders: The past is prologue. Nat. Rev. Drug Discov..

[44] Kwan J.A., Schulze C.J., Wang W., Leon H., Sariahmetoglu M., Sung M., Sawicka J., Sims D.E., Sawicki G., Schulz R. (2004). Matrix metalloproteinase-2 (MMP-2) is present in the nucleus of cardiac myocytes and is capable of cleaving poly (ADP-ribose) polymerase (PARP) in vitro. FASEB J..

[45] Lammerding J. (2011). Mechanics of the nucleus. Compr. Physiol..

[46] Green D.R., Llambi F. (2015). Cell Death Signaling. Cold Spring Harb. Perspect. Biol..

[47] Schweichel J.-U., Merker H.-J. (1973). The morphology of various types of cell death in prenatal tissues. Teratology.

[48] Galluzzi L., Vitale I., Aaronson S.A., Abrams J.M., Adam D., Agostinis P., Alnemri E.S., Altucci L., Amelio I., Andrews D.W. (2018). Molecular mechanisms of cell death: Recommendations of the Nomenclature Committee on Cell Death 2018. Cell Death Differ..

[49] Kroemer G., El-Deiry W.S., Golstein P., Peter M.E., Vaux D., Vandenabeele P., Zhivotovsky B., Blagosklonny M.V., Malorni W., Knight R.A. (2005). Classification of cell death: Recommendations of the nomenclature committee on cell death. Cell Death Differ..

[50] Czabotar P.E., Lessene G., Strasser A., Adams J.M. (2014). Control of apoptosis by the BCL-2 protein family: Implications for physiology and therapy. Nat. Rev. Mol. Cell Biol..

[51] Singh R., Letai A., Sarosiek K. (2019). Regulation of apoptosis in health and disease: The balancing act of BCL-2 family proteins. Nat. Rev. Mol. Cell Biol..

[52] Nicholson D.W., Thornberry N.A. (1997). Caspases: Killer proteases. Trends Biochem. Sci..

[53] Liu X., Zou H., Slaughter C., Wang X. (1997). DFF, a heterodimeric protein that functions downstream of caspase-3 to trigger DNA fragmentation during apoptosis. Cell.

[54] Sakahira H., Enari M., Nagata S. (1998). Cleavage of CAD inhibitor in CAD activation and DNA degradation during apoptosis. Nature.

[55] Enari M., Sakahira H., Yokoyama H., Okawa K., Iwamatsu A., Nagata S. (1998). A caspase-activated DNase that degrades DNA during apoptosis, and its inhibitor ICAD. Nature.

[56] Andrabi S.A., Dawson T.M., Dawson V.L. (2008). Mitochondrial and nuclear cross talk in cell death: Parthanatos. Ann. N. Y. Acad. Sci..

[57] David K.K., Andrabi S.A., Dawson T.M., Dawson V.L. (2009). Parthanatos, a messenger of death. Front. Biosci..

[58] Vorobjeva N.V., Chernyak B.V. (2020). NETosis: Molecular Mechanisms, Role in Physiology and Pathology. Biochemistry (Mosc.).

[59] Thiam H.R., Wong S.L., Wagner D.D., Waterman C.M. (2021). Cellular Mechanisms of NETosis. Annu. Rev. Cell Dev. Biol..

[60] Neubert E., Meyer D., Rocca F., Günay G., Kwaczala-tessmann A., Grandke J., Senger-sander S., Geisler C., Egner A., Schön M.P. (2018). Chromatin swelling drives neutrophil extracellular trap release. Nat. Commun..

[61] Sheshachalam A., Srivastava N., Mitchell T., Lacy P., Eitzen G. (2014). Granule protein processing and regulated secretion in neutrophils. Front. Immunol..

[62] Papayannopoulos V., Metzler K.D., Hakkim A., Zychlinsky A. (2010). Neutrophil elastase and myeloperoxidase regulate the formation of neutrophil extracellular traps. J. Cell Biol..

[63] Wang Y., Li M., Stadler S., Correll S., Li P., Wang D., Hayama R., Leonelli L., Han H., Grigoryev S.A. (2009). Histone hypercitrullination mediates chromatin decondensation and neutrophil extracellular trap formation. J. Cell Biol..

[64] Hamam H.J., Palaniyar N. (2019). Post-Translational Modifications in NETosis and NETs-Mediated Diseases. Biomolecules.

[65] Ruchaud S., Korfali N., Villa P., Kottke T.J., Dingwall C., Kaufmann S.H., Earnshaw W.C. (2002). Caspase-6 gene disruption reveals a requirement for lamin A cleavage in apoptotic chromatin condensation. EMBO J..

[66] (1990). Kenji Takahashi Calpain Substrate Specificity. Intracellular Calcium-Dependent Proteolysis.

[67] Chen K.W., Monteleone M., Boucher D., Sollberger G., Ramnath D., Condon N.D., von Pein J.B., Broz P., Sweet M.J., Schroder K. (2018). Noncanonical inflammasome signaling elicits gasdermin D–dependent neutrophil extracellular traps. Sci. Immunol..

[68] Li Y., Li M., Weigel B., Mall M., Werth V.P., Liu M. (2020). Nuclear envelope rupture and NET formation is driven by PKCα-mediated lamin B disassembly. EMBO Rep..

[69] Torabi B., Flashner S., Beishline K., Sowash A., Donovan K., Bassett G., Azizkhan-Clifford J. (2018). Caspase cleavage of transcription factor Sp1 enhances apoptosis. Apoptosis.

[70] Tewari M., Quan L.T., O’Rourke K., Desnoyers S., Zeng Z., Beidler D.R., Poirier G.G., Salvesen G.S., Dixit V.M. (1995). Yama/CPP32β, a mammalian homolog of CED-3, is a CrmA-inhibitable protease that cleaves the death substrate poly(ADP-ribose) polymerase. Cell.

[71] Kim B.J., Lee H. (2008). Caspase-mediated cleavage of importin-α increases its affinity for MCM and downregulates DNA synthesis by interrupting the binding of MCM to chromatin. Biochim. Biophys. Acta-Mol. Cell Res..

[72] Ubeda M., Habener J.F. (1997). The Large Subunit of the DNA Replication Complex C (DSEB/RF-C140) Cleaved and Inactivated by Caspase-3 (CPP32/YAMA) during Fas-induced Apoptosis. J. Biol. Chem..

[73] Huang Y., Nakada S., Ishiko T., Utsugisawa T., Datta R., Kharbanda S., Yoshida K., Talanian R.V., Weichselbaum R., Kufe D. (1999). Role for Caspase-Mediated Cleavage of Rad51 in Induction of Apoptosis by DNA Damage. Mol. Cell. Biol..

[74] Tang D., Kidd V.J. (1998). Cleavage of DFF-45/ICAD by Multiple Caspases Is Essential for Its Function during Apoptosis. J. Biol. Chem..

[75] Zhang Z., Larner S.F., Liu M.C., Zheng W., Hayes R.L., Wang K.K.W. (2009). Multiple alphaII-spectrin breakdown products distinguish calpain and caspase dominated necrotic and apoptotic cell death pathways. Apoptosis.

[76] Kenig S., Frangež R., Pucer A., Lah T. (2011). Inhibition of cathepsin L lowers the apoptotic threshold of glioblastoma cells by up-regulating p53 and transcription of caspases 3 and 7. Apoptosis.

[77] Vancompernolle K., Van Herreweghe F., Pynaert G., Van de Craen M., De Vos K., Totty N., Sterling A., Fiers W., Vandenabeele P., Grooten J. (1998). Atractyloside-induced release of cathepsin B, a protease with caspase-processing activity. FEBS Lett..

[78] Zhang D., Beresford P.J., Greenberg A.H., Lieberman J. (2001). Granzymes A and B directly cleave lamins and disrupt the nuclear lamina during granule-mediated cytolysis. Proc. Natl. Acad. Sci. USA.

[79] Lu H., Hou Q., Zhao T., Zhang H., Zhang Q., Wu L., Fan Z. (2006). Granzyme M Directly Cleaves Inhibitor of Caspase-Activated DNase (CAD) to Unleash CAD Leading to DNA Fragmentation. J. Immunol..

[80] Froelich C.J., Hanna W.L., Poirier G.G., Duriez P.J., D’amours D., Salvesen G.S., Alnemri E.S., Earnshaw W.C., Shah G.M. (1996). Granzyme B/Perforin-Mediated Apoptosis of Jurkat Cells Results in Cleavage of Poly(ADP-ribose) Polymerase to the 89-kDa Apoptotic Fragment and Less Abundant 64-kDa Fragment. Biochem. Biophys. Res. Commun..

[81] Pinkoski M.J., Heibein J.A., Barry M., Bleackley R.C. (2000). Nuclear translocation of granzyme B in target cell apoptosis. Cell Death Differ..

[82] McGinnis K.M., Gnegy M.E., Park Y.H., Mukerjee N., Wang K.K.W. (1999). Procaspase-3 and Poly(ADP)ribose polymerase (PARP) are calpain substrates. Biochem. Biophys. Res. Commun..

[83] Tremper-Wells B., Vallano M. (2005). Lou Nuclear calpain regulates Ca^2+^-dependent signaling via proteolysis of nuclear Ca2+/calmodulin-dependent protein kinase type IV in cultured neurons. J. Biol. Chem..

[84] Abe K., Takeichi M. (2007). NMDA-Receptor Activation Induces Calpain-Mediated β-Catenin Cleavages for Triggering Gene Expression. Neuron.

[85] Hirai S., Kawasaki H., Yaniv M., Suzuki K. (1991). Degradation of transcription factors, c-Jun and c-Fos, by calpain. FEBS Lett..

[86] Pariat M., Salvat C., Bebien M., Brockly F., Altieri E., Carillo S., Jariel-Encontre I., Piechaczyk M. (2000). The sensitivity of c-Jun and c-Fos proteins to calpains depends on conformational determinants of the monomers and not on formation of dimers. Society.

[87] Mao X., Yang S.-H., Simpkins J.W., Barger S.W. (2007). Glutamate receptor activation evokes calpain-mediated degradation of Sp3 and Sp4, the prominent Sp-family transcription factors in neurons. J. Neurochem..

[88] Kubbutat M.H., Vousden K.H. (1997). Proteolytic cleavage of human p53 by calpain: A potential regulator of protein stability. Mol. Cell. Biol..

[89] Nishinaka T., Fu Y.H.F., Chen L.I., Yokoyama K., Chiu R. (1997). A unique cathepsin-like protease isolated from CV-1 cells is involved in rapid degradation of retinoblastoma susceptibility gene product, RB, and transcription factor SP1. Biochim. Biophys. Acta-Gene Struct. Expr..

[90] Gößwein S., Lindemann A., Mahajan A., Maueröder C., Martini E., Patankar J., Schett G., Becker C., Wirtz S., Naumann-Bartsch N. (2019). Citrullination licenses calpain to decondense nuclei in neutrophil extracellular trap formation. Front. Immunol..

[91] Dhaenens M., Glibert P., Lambrecht S., Vossaert L., Van Steendam K., Elewaut D., Deforce D. (2014). Neutrophil Elastase in the capacity of the “H2A-specific protease”. Int. J. Biochem. Cell Biol..

[92] Yuste V.J., Moubarak R.S., Delettre C., Bras M., Sancho P., Robert N., D’Alayer J., Susin S.A. (2005). Cysteine protease inhibition prevents mitochondrial apoptosis-inducing factor (AIF) release. Cell Death Differ..

[93] Solary E., Eymin B., Droin N., Haugg M. (1998). Proteases, proteolysis, and apoptosis. Cell Biol. Toxicol..

[94] Nicholson D.W. (1999). Caspase structure, proteolytic substrates, and function during apoptotic cell death. Cell Death Differ..

[95] Nuñez G., Benedict M.A., Hu Y., Inohara N. (1998). Caspases: The proteases of the apoptotic pathway. Oncogene.

[96] Elmore S. (2007). Apoptosis: A Review of Programmed Cell Death. Toxicol. Pathol..

[97] Ishisaka R., Utsumi K., Utsumi T. (2002). Involvement of Lysosomal Cysteine Proteases in Hydrogen Peroxide-induced Apoptosis in HL-60 Cells. Biosci. Biotechnol. Biochem..

[98] Chow S.C., Weis M., Kass G.E., Holmstro T.H., Eriksson J.E., Orrenius S. (1995). Involvement of multiple proteases during Fas-mediated apoptosis in T lymphocytes. FEBS Lett..

[99] Schlegel J., Peters I., Orrenius S. (1995). Isolation and partial characterization of a protease involved in Fas-induced apoptosis. FEBS Lett..

[100] Zhivotovsky B., Wade D., Gahm A., Orrenius S., Nicotera P. (1994). Formation of 50 kbp chromatin fragments in isolated liver nuclei is mediated by protease and endonuclease activation. FEBS Lett..

[101] Momeni H.R. (2011). Role of Calpain in Apoptosis. Cell J..

[102] Hay Z.L.Z., Slansky J.E. (2022). Granzymes: The Molecular Executors of Immune-Mediated Cytotoxicity. Int. J. Mol. Sci..

[103] Chowdhury D., Lieberman J. (2008). Death by a Thousand Cuts: Granzyme Pathways of Programmed Cell Death. Annu. Rev. Immunol..

[104] Gao Y., Jiao Y., Gong X., Liu J., Xiao H., Zheng Q. (2023). Role of transcription factors in apoptotic cells clearance. Front. Cell Dev. Biol..

[105] Pizzorno M.C. (2001). Nuclear cathepsin B-like protease cleaves transcription factor YY1 in differentiated cells. Biochim. Biophys. Acta-Mol. Basis Dis..

[106] Zhang J.J., Zhu Y., Yang C., Liu X., Peng Y.P., Jiang K.R., Miao Y., Xu Z.K. (2016). Yin Yang-1 increases apoptosis through Bax activation in pancreatic cancer cells. Oncotarget.

[107] Deniaud E., Baguet J., Mathieu A.L., Pagès G., Marvel J., Leverrier Y. (2006). Overexpression of Sp1 transcription factor induces apoptosis. Oncogene.

[108] Zhu T., Zheng J.Y., Huang L.L., Wang Y.H., Yao D.F., Dai H. (2023). Bin Human PARP1 substrates and regulators of its catalytic activity: An updated overview. Front. Pharmacol..

[109] Fatokun A.A., Dawson V.L., Dawson T.M. (2016). Parthanatos: Mechanisms and therapeutic opportunities. Br. J. Pharmacol..

[110] Von Köckritz-Blickwede M., Goldmann O., Thulin P., Heinemann K., Norrby-Teglund A., Rohde M., Medina E. (2008). Phagocytosis-independent antimicrobial activity of mast cells by means of extracellular trap formation. Blood.

[111] Obata-ninomiya K., Karasuyama H., Djonov V., Eggel A. (2014). NADPH Oxidase–Independent Formation of Extracellular DNA Traps by Basophils. J. Immunol..

[112] Granger V., Faille D., Marani V., Noël B., Gallais Y., Szely N., Flament H., Pallardy M., Chollet-Martin S., de Chaisemartin L. (2017). Human blood monocytes are able to form extracellular traps. J. Leukoc. Biol..

[113] Chow O.A., von Köckritz-Blickwede M., Bright A.T., Hensler M.E., Zinkernagel A.S., Cogen A.L., Gallo R.L., Monestier M., Wang Y., Glass C.K. (2010). Statins Enhance Formation of Phagocyte Extracellular Traps. Cell Host Microbe.

[114] Wang H., Zhang Y., Wang Q., Wei X., Wang H., Gu K. (2021). The regulatory mechanism of neutrophil extracellular traps in cancer biological behavior. Cell Biosci..

[115] Metzler K.D., Goosmann C., Lubojemska A., Zychlinsky A. (2014). A Myeloperoxidase-Containing Complex Regulates Neutrophil Elastase Release and Actin Dynamics during NETosis. Cell Rep..

[116] Dhaenens M., Glibert P., Meert P., Vossaert L., Deforce D. (2015). Histone proteolysis: A proposal for categorization into ‘clipping’ and ‘degradation’. BioEssays.

[117] Salvesen G.S. (2020). NETosis occurs independently of neutrophil serine proteases. J. Biol. Chem..

[118] Jackson H.W., Defamie V., Waterhouse P., Khokha R. (2017). TIMPs: Versatile extracellular regulators in cancer. Nat. Rev. Cancer.

[119] Turk V., Stoka V., Turk D. (2008). Cystatins: Biochemical and structural properties, and medical relevance. Front. Biosci..

[120] Imre G., Berthelet J., Heering J., Kehrloesser S., Melzer I.M., Lee B.I., Thiede B., Dötsch V., Rajalingam K. (2017). Apoptosis inhibitor 5 is an endogenous inhibitor of caspase-2. EMBO Rep..

[121] Wendt A., Thompson V.F., Goll D.E. (2004). Interaction of Calpastatin with Calpain: A Review. Biol. Chem..

[122] Li H., Nishio K., Yamashita K., Hayakawa T., Hoshino T. (1995). Cell cycle-dependent localization of tissue inhibitor of metalloproteinases-1 immunoreactivity in cultured human gingival fibroblasts. Nagoya J. Med. Sci..

[123] Rudzinska-Radecka M., Frolova A.S., Balakireva A.V., Gorokhovets N.V., Pokrovsky V.S., Sokolova D.V., Korolev D.O., Potoldykova N.V., Vinarov A.Z., Parodi A. (2022). In Silico, In Vitro, and Clinical Investigations of Cathepsin B and Stefin A mRNA Expression and a Correlation Analysis in Kidney Cancer. Cells.

[124] Kopitar-Jerala N. (2013). Cysteine Proteinase Inhibitors in the Nucleus and Nucleolus in Activated Macrophages. Proteins of the Nucleolus: Regulation, Translocation, & Biomedical Functions.

[125] Lamort A.S., Hamon Y., Czaplewski C., Gieldon A., Seren S., Coquet L., Lecaille F., Lesner A., Lalmanach G., Gauthier F. (2019). Processing and Maturation of Cathepsin C Zymogen: A Biochemical and Molecular Modeling Analysis. Int. J. Mol. Sci..

[126] Turk B., Dolenc I., Lenarčič B., Križaj I., Turk V., Bieth J.G., Björk I. (1999). Acidic pH as a physiological regulator of human cathepsin L activity. Eur. J. Biochem..

[127] Young Choi K., Swierczewska M., Lee S., Chen X. (2012). Protease-Activated Drug Development. Theranostics.

[128] Verma S., Dixit R., Pandey K.C. (2016). Cysteine proteases: Modes of activation and future prospects as pharmacological targets. Front. Pharmacol..

